# On a Sensor Placement Methodology for Monitoring the Vibrations of Horizontally Excited Ground

**DOI:** 10.3390/s20071938

**Published:** 2020-03-30

**Authors:** Aneta Herbut, Jarosław Rybak, Włodzimierz Brząkała

**Affiliations:** Faculty of Civil Engineering, Wrocław University of Science and Technology, Wyb. Wyspiańskiego 27, 50-370 Wrocław, Poland; jaroslaw.rybak@pwr.edu.pl (J.R.); wlodzimierz.brzakala@pwr.edu.pl (W.B.)

**Keywords:** vibration monitoring, horizontal impact load, vibration pollution, measuring environmental variables, wave propagation

## Abstract

In this paper, the problem of optimal sensor arrangement during vibration monitoring is analysed. The wave propagation caused by horizontal excitation is investigated to predict the areas of the largest ground and structure response. The equations of motion for a transversally isotropic elastic medium with appropriate absorbing boundary conditions are solved using the finite element method (FlexPDE software). The possibility of an amplified soil medium response is examined for points located on the ground surface and at various depths. The results are presented in the form of a dimensionless vibration reduction factor, defined as the ratio of the peak particle velocity observed at the selected depth to the corresponding value observed at the ground surface. Significant amplifications (≈50%) can be observed below the ground surface, especially in the case of a weak layer below a stiff layer. The effect of vibration amplification is most significant near the boundary surface of two layers. For the points located on the ground surface, the greatest peak particle velocities are observed in the direction perpendicular to the load direction. However, the greatest vertical velocity component at the ground surface is observed in front of the applied force.

## 1. Introduction: The Importance of the Analysed Problem

Three major issues are related to the structural health monitoring of buildings, underground structures and other civil engineering structures in the case of dynamic excitation (traffic load or geotechnical works) [1,2,3,4]. The first issue is directly related to vibration sensors (accelerometers, interferometric sensors, and fibre optic sensors) and their sensitivity and applicability [2]. The second, not yet sufficiently analysed issue, relates to the sensor layout [5,6,7], data transmission [8] and ability to process the recorded signals in real time (as imposed vibrations can cause serious damage to the monitored structures [9]). The final problem, which has already been widely discussed and described in codes and recommendations, involves the threshold values and criteria adopted in current engineering practices (interpretation of monitoring results) [10,11,12,13,14,15]. This paper addresses the second problem noted, namely, the consequences of sensor layout. In this paper, suggestions on the proper sensor arrangement are proposed.

This paper concerns the ground and structure vibrations caused by horizontally oriented excitation. During the vibration monitoring of engineering structures, a very important issue is to choose appropriate sensor locations. As the measured values in the form of three velocity components (three orthogonal directions *x*, *y*, and *z*) are compared to threshold values given by special codes for vibration monitoring, it is crucial to locate sensors at points where the maximal soil/structure response can be observed [10,11,12,13,14,15]. The structure response should not be underestimated, and buildings must remain safe during dynamic excitation. Extensive comparison studies of threshold values according to different codes can be found in the literature [16,17,18,19,20]. Some general remarks on sensor arrangement are given in different codes and standards. Vibration monitoring for buildings should be based on data recorded at the foundation level and on different floors (usually the highest one) [10,11,12,13,14,15]. However, the problem is more complicated in the case of underground structures, for which vibration monitoring should be performed with sensors located directly on the structures [10,11,12,13,14,15]. This approach requires removing soil between the structure and ground surface, which is often not possible or even safe for the structure. In some practical applications of structural monitoring, sensor placement is based on engineering judgement, for example, sensor locations are chosen using previous experience. Notwithstanding, the sensor layout is crucial to the successful identification of the largest values of soil/structure response due to dynamic excitation, so a systematic approach is required [21]. Wave propagation is analysed in this paper to better understand the phenomenon of wave propagation in soil caused by horizontal forces. Based on the results obtained for numerical simulations, the areas of the largest ground and structure response are predicted. In this way, the authors can identify the best sensor (accelerometer) locations for the vibration control of both buildings and buried structures in the course of horizontal directional jacking. Specifically, the effect of periodic horizontal excitation at depth is studied for different sensor system designs to determine how to predict vibration amplitudes below the ground surface based on analyses conducted with data from the appropriate points located on the ground surface.

**Figure 1:**
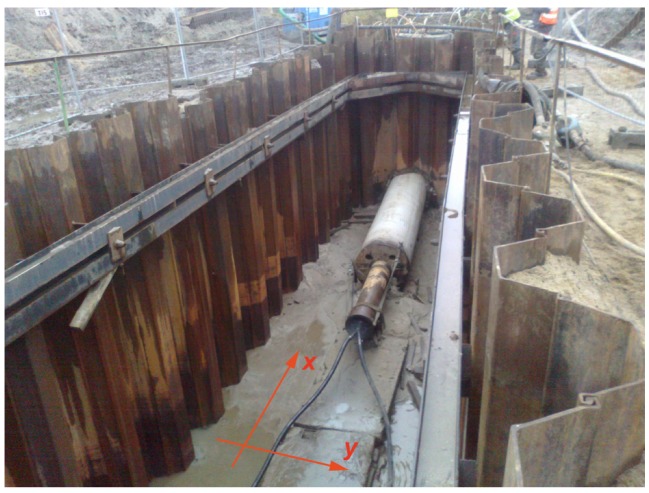
Impact installation of a pipe segment.

**Figure 2:**
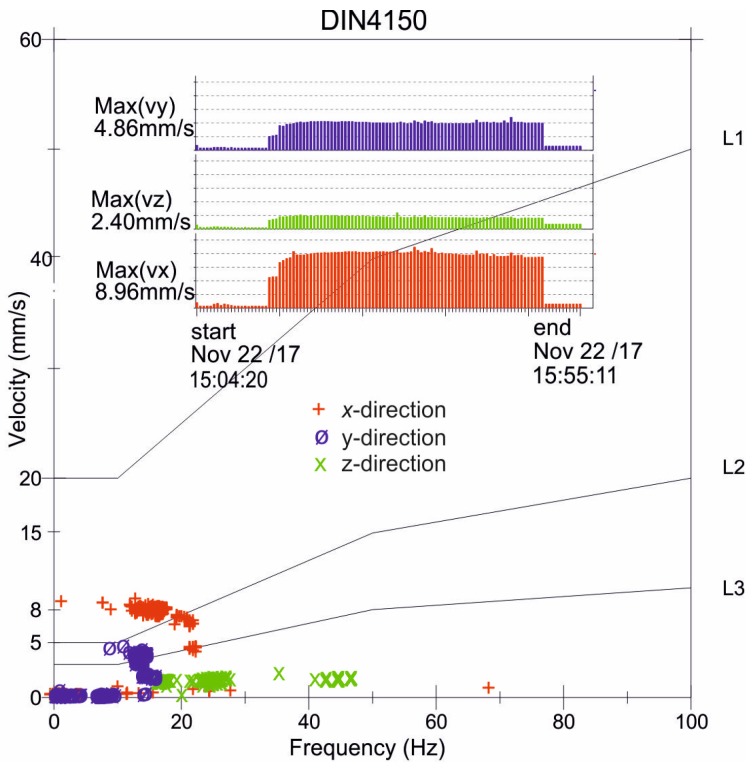
Results of the vibration monitoring of the ground surface during the impact installation of a pipe segment (**L1**—industrial buildings, **L2**—dwellings, **L3**—buildings sensitive to vibration).

From an engineering perspective, horizontal excitation is very important, especially for some geotechnical works, such as the dynamic installation of a pipe segment using underground trenchless technology (Figure 1). Many attempts to measure the influence of underground works on existing infrastructure have been made and reported in the course of large infrastructural projects, mainly including tunnelling works [22,23,24]. The impact of dynamic guided jacking is often considered “less important”, which is why there is a lack of research on this type of excitation. However, such an excitation can cause vibration amplitudes greater than the threshold values presented in standards for vibration control [10,11,12,13,14,15]. This type of excitation and the possible damage it causes are very important, especially in cases when geotechnical works are implemented in the vicinity of existing pipelines that should soon be replaced due to their poor technical condition but must still be operated. Some selected results of vibration monitoring, carried out by the authors during a pipe installation process, are presented in Figure 2. Notably, 3D sensors were located at distances of 8 m and 12 m in the *x*-direction and *y*-direction, respectively, from the vibration source (Figure 1). The sensors were located near the ground surface. The maximum velocity component was measured in the horizontal direction (*x*-direction) of excitation (Figure 2). A value of close to 9 mm/s exceeds the threshold values given in the DIN4150 Standard for both vibration-sensitive structures (L3) and dwellings (L2) (Figure 2). In the presented case, the velocity amplitudes were much larger closer to the vibration source. The conclusion that can be drawn from these measurements is that vibrations caused by horizontal excitation can cause serious damage to many types of structure (Figure 2). The issue of the rational arrangement of sensors in the case of horizontally excited vibrations is of great importance but it is still not widely analysed nor described. Note that criteria for vibration monitoring during geotechnical works are more restrictive for the smaller values of the excitation frequencies (1–10 Hz), bearing in mind the natural frequencies of buildings (Figure 2). Threshold values of velocity amplitudes are established with the assumption of possible structural resonance. 

The case of horizontal dynamic excitation has rarely been investigated in the literature compared to vibrations excited by vertically oriented forces. The case of vertical dynamic excitation is widely described in the literature, and the wave propagation phenomenon caused by it is clear and easy to predict [16,17,25,26,27,28,29]. In the case of a vertical force applied to the ground surface, the Rayleigh wave is dominant. This wave propagates as a source of vibration outwards in a cylindrical wave front. The horizontal amplitude of the Rayleigh wave has a maximum value at the ground surface and is strongly attenuated below the surface. However, the vertical displacement component first increases to the maximum value, observed at a depth of approximately 0.076*λ*_R_, where *λ*_R_ is the wavelength of the Rayleigh wave. After reaching the maximum value, the vertical vibration component decreases to zero, observed at nearly 2*λ*_R_ [25]. The vibration intensity is attenuated with increasing distance from the vibration source. This effect is caused by two different factors: Material and geometrical damping [25,30]. Geometrical damping occurs as a result of the expansion of the wave front. Material damping is closely connected with the physical parameters of the soil medium.

In the case of pipe installation, an excitation force occurs at the excavation site (Figure 1), which influences the wave propagation phenomena. Upon contact with such an impediment in the soil (excavation site), a wave is reflected and refracted, and new body waves appear. The issue of practical importance is determining how to predict the location of the maximum soil response (displacement and velocity). How can sensors be arranged during monitoring to measure the maximum soil response? Note that this question is important not only for points located on the ground surface, but also for points below it. The following question emerges: What range of soil response amplification can be expected below the ground surface compared to that measured on the ground surface? This issue is important in the case of underground structures when collecting measurements on the structure is impossible. In the case of vertical excitation, wave attenuation below the ground surface was examined by Ekanayake et al. [17] in the case of pile driving. During the first steps of pile driving, when the excitation force acts close to the ground surface, wave attenuation was observed below the ground surface. This conclusion is obvious, because in this case the Rayleigh wave is dominant. However, for later steps of pile driving, when the excitation force acts at a deeper level, some vibration amplitude amplification was observed at shallow depths [17]. The observations made by Ekanayake et al. [17] for harmonic excitation correspond to the results presented by Masoumi et al. [24] for the case of dynamic impact loads. Masoumi et al. [31] presented extensive numerical studies of wave propagation in soil caused by both vibrations and impact pile driving. The coupled finite element method (FEM)-boundary element method was implemented to solve the linear equations of motion for a layered medium. For each analysed case, the maximum observed displacement/velocity/acceleration values may appear below the ground surface, especially when the excitation force is not directly applied to the ground surface [17,31]. This conclusion shows that data from vibration monitoring on the ground surface are not always representative enough to predict the maximum underground structure response to a dynamic load. The aim of this paper is to examine the scale of vibration amplification below the ground surface that can be observed for horizontally oriented dynamic loads, which has not been widely described in the literature [32]. Moreover, the propagation of waves in both horizontal directions, that is, along the excitation line and along the perpendicular line, is studied. The potential danger to a structure depends not only on the distance from the vibration source, but also on the orientation angle [33].

**Figure 3:**
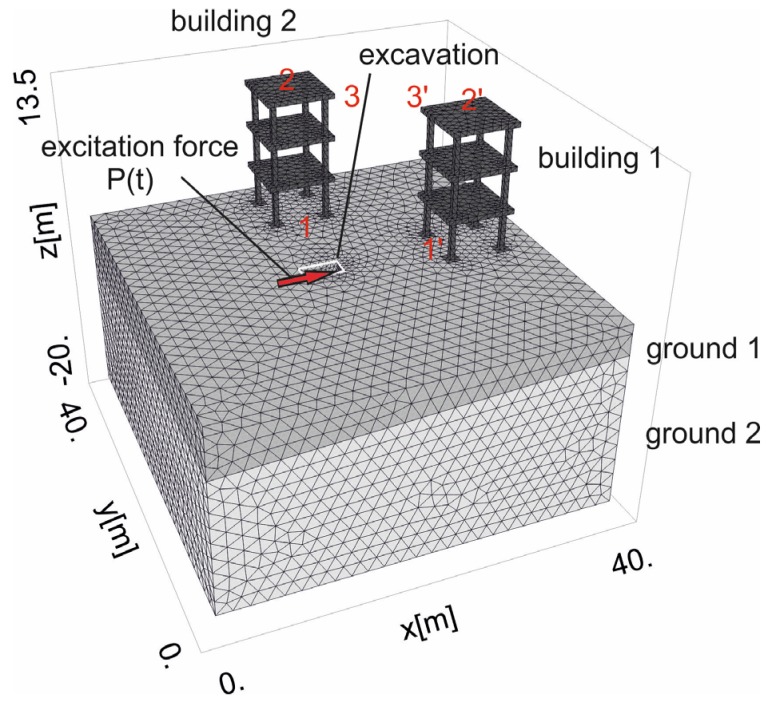
Considered domain of the numerical model.

In this paper, the authors perform numerical analyses for a soil medium with heterogeneous ground conditions (Figure 3). Two structure models are located at different points to determine in which area a more dangerous situation will occur. In Section 4.1, the wave propagation caused by horizontal excitation is examined for homogenous ground conditions. The results are presented for points located on the ground surface, and the areas associated with the greatest soil and structure responses are identified. In Section 4.2, the analyses are widened to points located below the ground surface. The areas of greatest velocity amplification are shown for horizontal cross-sections parallel to the ground surface but located at selected depths below it. The results are compared to the vibration amplitudes obtained for the corresponding points located directly on the ground surface. Heterogenous ground conditions are studied, for which prediction of the greatest possible amplification of the soil response is less intuitive.

In this paper, the results of the soil response are presented in the form of velocity components in three orthogonal directions (*x*, *y*, and *z*). According to different European standards [12,14,16,20], the *PPV* (peak particle velocity) can be calculated. The *PPV* describes the intensity of ground vibration and can be used to determine the level of structural safety by comparison with threshold values. As the particle velocity in the medium is proportional to the strains generated in the soil, velocities are the most common unit of the soil response intensity [16,17]. Four standard approaches can be used to determine the *PPV*. The most common approach is to determine the peak velocity value based on measurements in three directions. This concept is applied in the present study. In other approaches, the PPV can be obtained based on the peak value only in the vertical direction, the square root of the sum of the squares of peak values in each direction (*SRSS*), and the peak value of the true vector sum of velocities in three directions (*TVS*) [16,17]. According to Athanasopoulos and Pelekis [16], the *PPV* based on component particle velocities may be up to 25% lower than the *TVS*. In contrast, the *SRSS* may be 50% greater than the *TVS*.

## 2. Mathematical and Numerical Models

A 3D elastic linear continuum is considered that fills up a (theoretical) half-space with a horizontal boundary at *z* = 0. Depending on the geological situation, two cases were considered: The subsoil is either homogeneous or heterogeneous (layered). In each layer, the material was assumed to be homogeneous and transversally isotropic. The differential equations of motion of the soil medium can be obtained by summing the forces acting on a soil element [34]:

(1) $$\begin{array}{c} \sum P_{x}=0:\;\frac{\partial \sigma_{x}}{\partial x}+\frac{\partial \tau_{yx}}{\partial y}+\frac{\partial \tau_{zx}}{\partial z}=\rho \frac{\partial^{2}u_{x}}{\partial t^{2}}\\ \sum P_{y}=0:\;\frac{\partial \tau_{xy}}{\partial x}+\frac{\partial \sigma_{y}}{\partial y}+\frac{\partial \tau_{zy}}{\partial z}=\rho \frac{\partial^{2}u_{y}}{\partial t^{2}}\\ \sum P_{z}=0:\;\frac{\partial \tau_{xz}}{\partial x}+\frac{\partial \tau_{yz}}{\partial y}+\frac{\partial \sigma_{z}}{\partial z}=\rho \frac{\partial^{2}u_{z}}{\partial t^{2}}, \end{array}$$

where *u_x_*, *u_y_* and *u_z_* are the displacements in the *x*-, *y*- and *z*-directions, respectively; $\sigma_{x}$, $\sigma_{y}$, $\sigma_{z}$, $\tau_{xy}=\tau_{yx}$, $\tau_{xz}=\tau_{zx}$, and $\tau_{yz}=\tau_{zy}$ are the normal and shear stresses; and ρ is the soil density. The equations for strains and rotations in terms of the displacements are as follows: $\varepsilon_{x}=\partial u_{x}/\partial x$, $\varepsilon_{y}=\partial u_{y}/\partial y$, $\varepsilon_{z}=\partial u_{z}/\partial z$, $\gamma_{xy}=\partial u_{y}/\partial x+\partial u_{x}/\partial y$, $\gamma_{xz}=\partial u_{z}/\partial x+\partial u_{x}/\partial z$, and $\gamma_{yz}=\partial u_{z}/\partial y+\partial u_{y}/\partial z$. For a linear viscoelastic material with a horizontal (*xy*) plane of isotropy, the stresses are related to the displacements by the following relations [34,35]:

(2) $$\begin{array}{c} \left[\begin{array}{c} \begin{array}{c} \begin{array}{c} \sigma_{x}\\ \sigma_{y}\\ \sigma_{z} \end{array}\\ \tau_{yz}\\ \tau_{xz} \end{array}\\ \tau_{xy} \end{array}\right]={\left[\begin{array}{cccccc} D_{11} & D_{12} & D_{13} & 0 & 0 & 0\\ D_{12} & D_{11} & D_{13} & 0 & 0 & 0\\ D_{13} & D_{13} & D_{33} & 0 & 0 & 0\\ 0 & 0 & 0 & D_{55} & 0 & 0\\ 0 & 0 & 0 & 0 & D_{55} & 0\\ 0 & 0 & 0 & 0 & 0 & D_{66} \end{array}\right]}^{-1}\left[\begin{array}{c} \begin{array}{c} \begin{array}{c} \varepsilon_{x}\\ \varepsilon_{y}\\ \varepsilon_{z} \end{array}\\ \gamma_{yz}\\ \gamma_{xz} \end{array}\\ \gamma_{xy} \end{array}\right]+\\ tr{\left[\begin{array}{cccccc} D_{11} & D_{12} & D_{13} & 0 & 0 & 0\\ D_{12} & D_{11} & D_{13} & 0 & 0 & 0\\ D_{13} & D_{13} & D_{33} & 0 & 0 & 0\\ 0 & 0 & 0 & D_{55} & 0 & 0\\ 0 & 0 & 0 & 0 & D_{55} & 0\\ 0 & 0 & 0 & 0 & 0 & D_{66} \end{array}\right]}^{-1}\cdot \frac{\partial }{\partial t}\left[\begin{array}{c} \begin{array}{c} \begin{array}{c} \varepsilon_{x}\\ \varepsilon_{y}\\ \varepsilon_{z} \end{array}\\ \gamma_{yz}\\ \gamma_{xz} \end{array}\\ \gamma_{xy} \end{array}\right] \end{array}$$

where *D*_11_ = 1/*E_x_*, *D*_12_ = −*ν_x_*/*E_x_*, *D*_13_ = −*ν_z_*/*E_z_*, *D*_33_ = 1/*E_z_*, *D*_55_ = 1/*G_xz_*, and *D*_66_ = 2(1 + *ν_x_*)/*E_x_* [26]; *E_x_ = E_y_* is the Young’s modulus in the plane of isotropy; *E_z_* is the Young’s modulus in the plane perpendicular to the plane of isotropy; *ξ* is the damping factor (“relaxation time” *tr* = 2*ξ*/*ω; ω* is the excitation frequency); *ν_x_* = *ν_y_*, and *ν_z_* are the Poisson ratios in the plane of isotropy and in the plane perpendicular to the plane of isotropy; and $G_{xz}\;$ is the shear modulus in the plane perpendicular to the plane of isotropy. Zero initial conditions were assumed in the numerical investigations. To avoid wave reflection at the boundary, when using the standard FEM approach, absorbing viscous boundary conditions were assumed [36,37]. The normal and shear stress components for virtual dampers “fixed” on the right (along coordinate *x* = 40 m in Figure 3) and left (along coordinate *x* = 0 in Figure 3) boundaries can be expressed as *σ*_x_ = *aρV*_x_*v_x_*, *τ_xy_* = *bρV_xy_v_y_*, *τ_xz_* = *bρV_xz_v_z_*, where *v_x_*, *v_y_*, *v_z_* are the velocities in the *x*-, *y*- and *z*-directions, respectively; *a* and *b* are parameters introduced to improve the wave absorption at the boundaries (*a* = 1 and *b* = 0.25 [36,37]); *V*_x_ denotes the P-wave velocity; and *V*_xy_ and *V*_xz_ denote the S-wave velocities. The absorbing boundary conditions for the near and far edges of the analysed domain (along coordinate *y* = 0 and *y* = 40 m in Figure 3) were assumed to be in the similar form. Additionally, displacement components were assumed to be zero at the bottom edge of the investigated region.

The numerical analyses of the presented cases were performed using FlexPDE Professional V6 software based on the FEM (www.pdesolutions.com). A single finite element in the form of a four-node linear tetrahedron with three degrees of freedom at each node was assumed. Extensive convergence studies were performed during calculations for both the boundary location and the number of finite elements assumed. For the boundary located 20 m from the vibration source, part of the reflected energy does not influence the final solution. Different numerical models were assumed before the final results were obtained. It was verified that the number of nodes (55,517) and the number of unknowns (333,102) used during calculations were sufficient to obtain convergence in numerical simulations. For instance, the relative difference between velocity components at the selected point (Point 1) for two numerical models (first model: Number of nodes 55,517 (333,102 unknowns); second model: Number of nodes 64,220 (384,720 unknowns)) was approximately 0.82% for the vertical velocity component *z*, approximately 0.24% for the horizontal velocity component *x*, and approximately 0.27% for the horizontal velocity component *y*.

**Figure 4:**
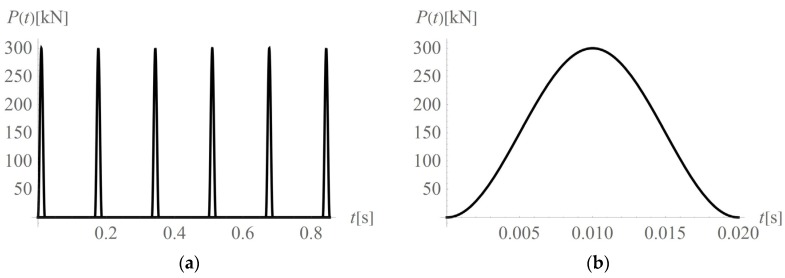
Vibration source; (**a**) sequence of impacts in the time domain; (**b**) first impact.

A haversine load of amplitude *A* = 300 kN (MAXK13OS equipment) and duration *d* = 0.02 s was applied to the ground surface at the excavation site (Figure 3 and Figure 4, [29,38]). The frequency of the excitation force is *f* = 6 Hz (Figure 4a). For each separate *i*th impact, the function *P_i_*(*t*) can be described as

(3) $$\begin{array}{c} P_{i}\left(t\right)=0.5A\left(1-\cos\left(2\pi \left(t-t_{b,i}\right)/\left(t_{e,i}-t_{b,i}\right)\right)\right)\cdot \left(H\left(t-t_{b,i}\right)-H\left(t-t_{e,i}\right)\right),\\ P\left(t\right)=\overset{n}{\underset{i=1}{\sum }}P_{i}\left(t\right), \end{array}$$

where H(*t*) is the Heaviside function, *t*_b,*i*_ (*t*_b,*I*_ = (*i* – 1)*T*) is the time when the machine starts working, *t*_e,*i*_ (*t*_e,*i*_= *t*_b,*I*_ + *d*) is the time when the machine stops working, and *n* is the number of impacts considered.

## 3. Soil Characteristics

The dynamic soil parameters of a transversally isotropic, two-layered half-space are presented in Table 1 [39,40,41]. Three different cases of ground conditions are taken into account in this paper. For the first case, homogenous soil characteristics are assumed (Case 1 in Table 1). In the second case, the dynamic elastic moduli for the first layer are assumed to be greater than those for the second layer (Case 2 in Table 1). In the third case, the opposite situation is analysed (Case 3 in Table 1). Additionally, the following soil characteristics remain the same for each layer: *ν_x_* = 0.301, *ν_z_* = 0.182, *ρ* = 2000 kg/m^3^, and *ξ* = 1%. The boundary surface between the two layers is inclined in one direction (*x*-direction) and defined by the points P_1_ (0, 0, −5.5 m), P_2_ (40 m, 0, −3.3 m), and P_3_ (40 m, 40 m, −3.3 m). Different types of soil layering were examined before the final selection of three cases presented in Table 1. The idea was to investigate the worst possible case of soil layering, which results in the most significant amplification of the dynamic response being below the ground surface. During investigations, it was found that for the large differences between elastic parameters of two first layers, the effect of wave reflection from the boundary surface is more visible. Two buildings with the same three-story structure were taken into account (building 1 and building 2 in Figure 3); both were located 10 m from the vibration source *P*(*t*) in perpendicular directions from *P*(*t*) (Figure 3). The square plates in each story were 10 m wide and 0.5 m thick. Each story was 4 m high. The concrete square columns were 0.8 m wide, and the concrete square foundation footings were 3 m wide and 0.8 m thick. The dynamic parameters of the concrete elements were as follows: *G_xz_* = 12.2 GPa, *E_x_ = E_y_* = 27 GPa, *ν_x_ = ν_y_* = 0.167, *ρ* = 2500 kg/m^3^, and *ξ* = 1% [40].

## 4. Results and Discussion

To investigate the level of vibration amplitude reduction below the ground surface, the nondimensional velocity amplitude reduction factor (*VRF_xyz_*) is introduced. For each selected point on a surface parallel to the ground surface, the maximum absolute value of the observed velocity components in the three directions is compared to that measured on the ground surface.

(4) $$VRF_{xyz}=PPV_{h}/PPV_{ho}=\mathrm{Max}\left(\left|v_{x,h}\right|,\left|v_{y,h}\right|,\left|v_{z,h}\right|\right)/\mathrm{Max}\left(\left|v_{x,ho}\right|,\left|v_{y,ho}\right|,\left|v_{z,ho}\right|\right).$$

To calculate *VRF_xyz_*, the maximum/minimum values of the three velocity components (*v_x,h_*, *v_y,h_*, *v_z,h_*) must be calculated for points located at the same depth (h) below the ground surface. For a selected point, *PPV_h_* is compared to the maximum/minimum observed velocity component *PPV_h_*_o_ calculated at a similar point (same *x* and *y*) located on the ground surface (*h*_o_ = 0). For each considered depth *h*, the calculations are made for 200 uniformly distributed points. The time considered is 6*T*, where *T* is the period of the excitation force. Please note that *PPV* is a commonly used value to predict the possibility of structural damage during dynamic excitation. The threshold values proposed in different standards for vibration control [10,11,12,13,14,15,16] are also presented in the form of *PPV* (4), which is why this factor is used in the paper.

### 4.1. The Maximum Soil Response Measured on the Ground Surface

The application of a dynamic load to the ground surface generates two types of waves that propagate outwards as sources of vibration. The first wave type includes body waves (P-waves, S-waves), which travel into the medium with a hemispherical wave front. Since P-waves are the fastest to propagate, they arrive first at a selected point on the ground surface, and are followed by S-waves. The attenuation (geometrical damping) of these two types of body waves is similar. In general, for a point situated below the ground surface, the vibration amplitudes decrease according to the function *r*^−1^, where *r* is the spatial distance from the point in the half-space to the vibration source [25,26,27]. For both body waves, the wave attenuation at points located on the ground surface can be described by the function *r*^−2^. In addition to body waves, surface waves are also generated. Surface waves propagate as a source of vibration outwards with a cylindrical wave front. The velocity of a surface wave is slightly lower than that of an S-wave and much lower than that of a P-wave. Thus, the surface waves arrive at the selected point on the ground surface as the final waves. However, the attenuation of surface waves is lower than that of body waves and can be described by the function *r*^−0.5^; this is why surface waves are the most dangerous factors in the case of an earthquake or manmade excitation. According to Kramer [26] and Towhata [27], surface waves transmit up to 67% of the total energy in the case of harmonic vertical excitation applied to the ground surface. The wave propagation in soil due to vertical excitation is widely described in the literature and is easy to predict [25,26,27]. The situation is more complicated and not as obvious in the case of horizontal excitation, especially when a load is not directly applied to the ground surface but acts at an excavation site. The aim of this section is to determine which region on the ground surface the maximum soil response can be expected.

**Figure 5:**
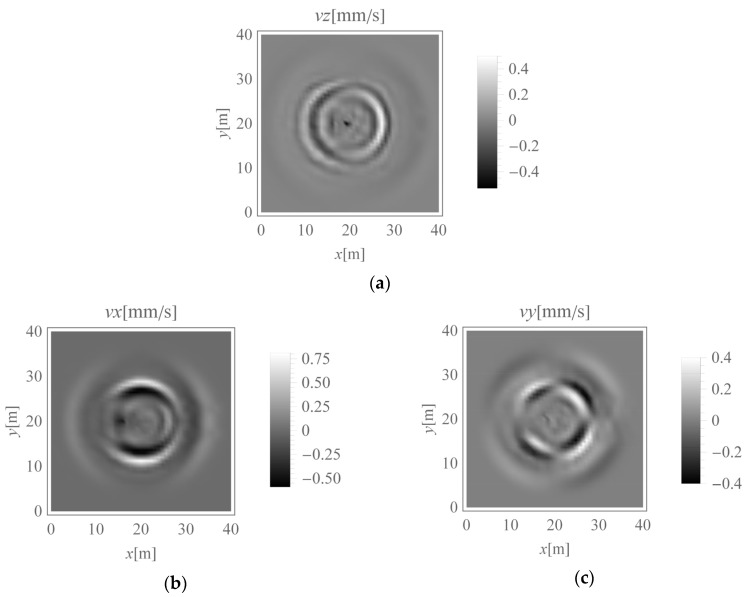
Velocity components in three directions under homogeneous ground conditions (*t* = 0.08 s): (**a**) *z*-direction, (**b**) *x*-direction, (**c**) *y*-direction.

**Figure 6:**
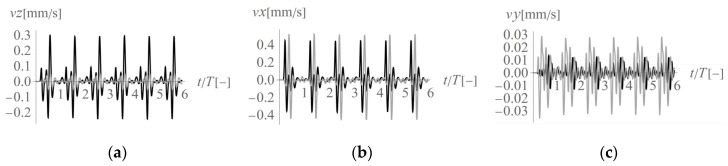
Three components of the velocity vector at Point 1 (1’) (Figure 3) under homogeneous ground conditions: (**a**) *z*-component, (**b**) *x*-component, (**c**) *y*-component; black line—building 1, grey line—building 2.

**Figure 7:**
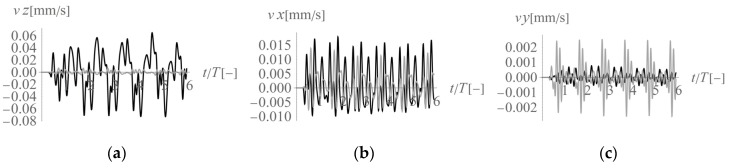
Three components of the velocity vector at Point 2 (2’) (Figure 3) under homogeneous ground conditions: (**a**) *z*-component, (**b**) *x*-component, (**c**) *y*-component; black line—building 1, grey line—building 2.

**Figure 8:**
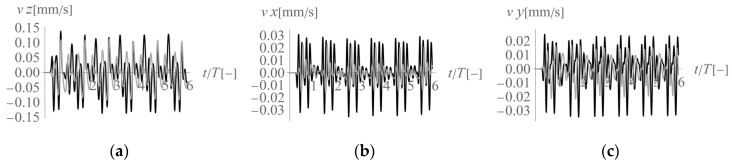
Three components of the velocity vector at Point 3 (3’) (Figure 3) under homogeneous ground conditions: (**a**) *z*-component, (**b**) *x*-component, (**c**) *y*-component; black line—building 1, grey line—building 2.

In the first analysed example, homogenous soil conditions are assumed according to Table 1 (Case 1). A periodic impact load is applied at the excavation site to the bottom of the vertical wall at a depth of *z* = −1 m (Figure 1 and Figure 3). The results are presented in the form of velocity components in three directions (*v_x_*, *v_y_*, *v_z_*) for points located on the ground surface (Figure 5) and for some characteristic points selected on both structures (Figure 3 and Figure 6, Figure 7 and Figure 8). To compare the results, two structures with the same geometrical features are taken into account (Figure 3). Both structures are located at the same distance from the vibration source but in different directions. Building 1 is located along the line of the dynamic load. Building 2 is located in the direction perpendicular to the excitation force direction. Both buildings are located in areas where the largest soil response can be observed (Figure 5). The results for the structures are presented in the form of velocity components in the time domain for three characteristic points usually selected for vibration monitoring according to the different European standards [10,11,12,13,14,15]. Point 1 is located at the foundation level and close to the vibration source. Point 2 is located in the middle of the highest floor. Point 3 is located on the highest floor in the corner. Both observation points (Point 2 and Point 3) are selected according to European standards requirements, in order to predict the possibility of resonant amplification of the whole structure or the selected structural element. Moreover, few cycles of excitation are taken into account to observe if resonant vibration can be observed in the analysed cases (Figure 6, Figure 7 and Figure 8). The details of the observation point locations are presented in Figure 3. In Figure 5, wave propagation in the soil caused by horizontal impulse excitation is presented for the selected point in time. The results are presented for the points located on the ground surface in the form of three components of the velocity vector *v_x_*_,_ *v_y_*, and *v_z_* (Figure 5a—*z*-direction; Figure 5b—*x*-direction; Figure 5b—*y*-direction). During the vibration monitoring of structures, similar results are obtained, with three velocity components for each measured point. Figure 5 shows the region in which the maximal soil response can be expected. For example, the vertical component has the largest value at *y* ~ 20 m (Figure 5a) along the direction of the applied load. This component has the greatest value near the point *x* ~ 25 m and *y* ~ 20 m, where the lightest and darkest colours on the graph appear (Figure 5a). Additionally, the maximal value of the horizontal velocity component appears in the perpendicular direction along the *x* ~ 20 m axis (Figure 5b). During the vibration monitoring of structures, the worst case must be predicted to arrange the sensors. Figure 5 shows the area in which the risk of structural damage is the largest. Based on this graph, the locations of building 1 and building 2 were chosen (see Figure 3) as the dynamic kinematic excitation is greatest there.

As the horizontal force acting along the *x*-axis is located in the middle of the analysed region, both surface and body waves appear. Greater velocity component values can be observed on the right side of the analysed region (*x* > 20 m, in front of the excited location) than the left side (*x* < 20 m, behind the excited location). The force is applied to the right wall of the excavation site (Figure 3). Note that no obvious symmetries about the *y* = 20 m axis are observed in Figure 5 because of the effects of the dynamic soil-structure interaction (presence of building 2). The excavation site is a barrier in the wave transmission path during wave propagation and on the left side of the applied force. Upon contact with the obstacle, a wave is reflected and refracted; therefore, the effect of wave energy scattering can be observed. Thus, the velocity amplitudes on the left side of the analysed region (*x* < 20 m) are smaller than those on the right side (Figure 5a–c). In Figure 5, the maximum velocity component is associated with the horizontal direction *x*, that is, the component *v_x_* dominates, especially in the middle of the analysed region (for *x* ~ 20 m). In this region, the amplification of the soil response in the *x*-direction is caused by both Rayleigh waves and S-waves because they propagate with similar velocities. However, in the other direction, in the middle of the analysed region (along the coordinate *y* = 20 m), slightly lower *x*-direction velocity values appear. In this case, amplification is caused by P-waves. As P-waves propagate with higher velocity than S-waves, the soil response in the *x*-direction (for building 1) can be observed before a similar effect is seen in the *y*-direction (for building 2) (Figure 6b).

In conclusion, according to different European standards for vibration control, vibration monitoring must be performed both at the foundation level and on the structure. The presented analyses show that the maximum velocity component value observed at the foundation level appears in the direction perpendicular to the horizontal excitation force direction in the middle of the considered region (along *x* = 20 m). In the case of the measurements made at the foundation level, the horizontal component in the *x*-direction is dominant (Figure 5 and Figure 6). The smaller the distance to the excitation force, the higher the maximum observed value. For both considered structures, the maximum observed velocity components are similar and are the velocity components in the horizontal *x*-direction (27% greater for building 2 compared to that for building 1). This phenomenon can be caused by the fact that in the region perpendicular to the excitation force (building 2), two waves propagate together, as they have similar velocities (S-wave and Rayleigh wave). The vibration effect is amplified in this case. In regions in front of the excitation force (building 1), the maximum vibrations are caused only by the P-wave (Figure 6b), which is why the effect is less significant. However, the vertical velocity component measured at the foundation level (Point 1) is six times greater for building 1, which is located in front of the excitation force (along *y* = 20 m), than for building 2 (Figure 6a). This observation should be taken into account, especially in the case of ceilings or rooves that are sensitive to vertical excitation. In such cases, sensors should also be located on building 1 to measure the structural response and avoid the resonant vibrations caused by vertical excitation. In the presented example, vibration amplification was not observed on the structure (Figure 7 and Figure 8). The calculated velocity components are below the threshold values given by the different European standards [10,11,12,13,14,15]. However, the possibility of resonant amplification on structures is possible, especially in the case of low excitation frequencies.

### 4.2. The Maximum Soil Response Measured below the Ground Surface

The aim of this section is to investigate how the maximum velocity component changes below the ground surface. This issue is important during vibration monitoring, especially for underground structures (tunnels, pipelines, etc.). According to German Standard DIN4150 [12], in the case of pipelines, sensors should be located directly on the pipe. However, placing a sensor on buried structures is usually complicated. Moreover, even if part of the structure can be exposed, this approach is not always safe. The monitoring results can be unrepresentative because the conditions of the soil-structure interaction are changed. The structural dynamic response is usually greater when the structure is not surrounded by soil. The results presented herein show how to correct the values measured on the ground surface to predict the maximum velocities below the ground surface.

In the case of vertical excitation directly applied to the ground surface, the attenuation effect on a surface wave below the ground is well known and has been widely explained in the literature [25,26]. For the horizontal displacement component, the values observed below the ground surface are always smaller than those measured on the ground surface. For the vertical displacement component, a certain small amplification (5%–10%) may appear at shallow depths [25]. For points located at greater depths, only the attenuation effect can be observed. This phenomenon has been noted when a vertical excitation force is directly applied to the ground surface and the Rayleigh wave is dominant. In the case of horizontally oriented excitation, the situation is not as obvious, as body waves (S-waves and P-waves) are more significant compared to those in the case of vertical excitation. When an additional excitation force occurs at an excavation site, the possibility of vibration amplification below the ground surface is more probable.

**Figure 9:**
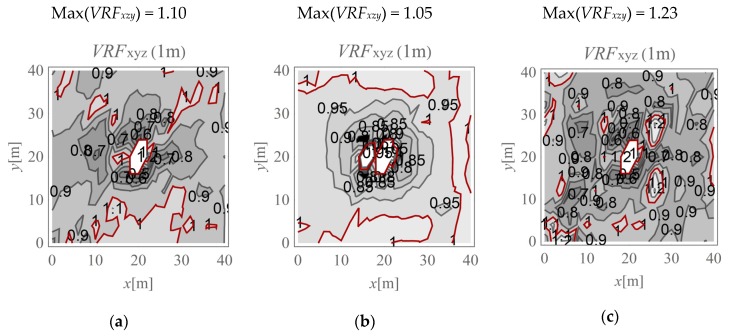
*VRF_xyz_* for the horizontal surface located at a depth of 1 m; (**a**) *G_xz_*_,1_ = *G_xz_*_,2_; (**b**) *G_xz_*_,1_ = 10*G_xz_*_,2_; (**c**) *G_xz_*_,2_ = 10*G_xz_*_,1_.

**Figure 10:**
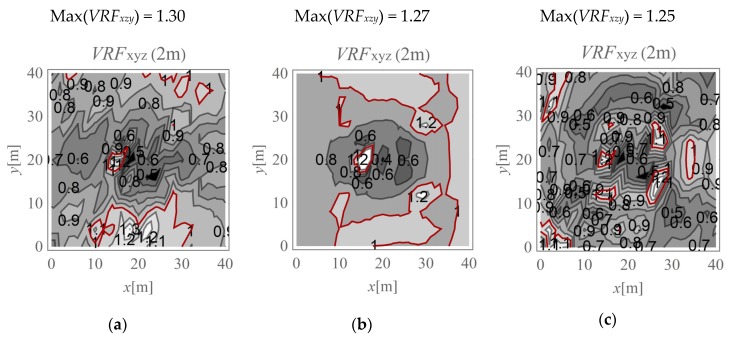
*VRF_xyz_* for the horizontal surface located at a depth of 2 m; (**a**) *G_xz_*_,1_ = *G_xz_*_,2_; (**b**) *G_xz_*_,1_ = 10*G_xz_*_,2_; (**c**) *G_xz_*_,2_ = 10*G_xz_*_,1_.

**Figure 11:**
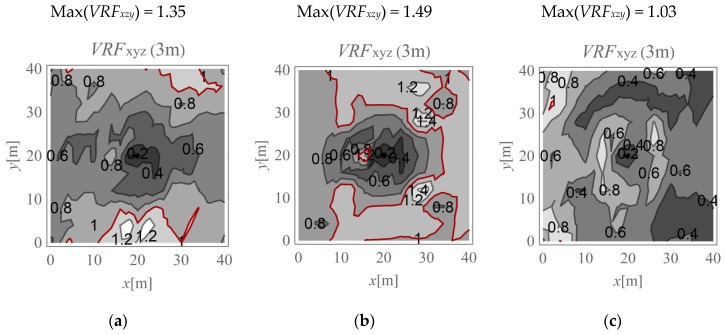
*VRF_xyz_* for the horizontal surface located at a depth of 3 m; (**a**) *G_xz_*_,1_ = *G_xz_*_,2_; (**b**) *G_xz_*_,1_ = 10*G_xz_*_,2_; (**c**) *G_xz_*_,2_ = 10*G_xz_*_,1_.

**Figure 12:**
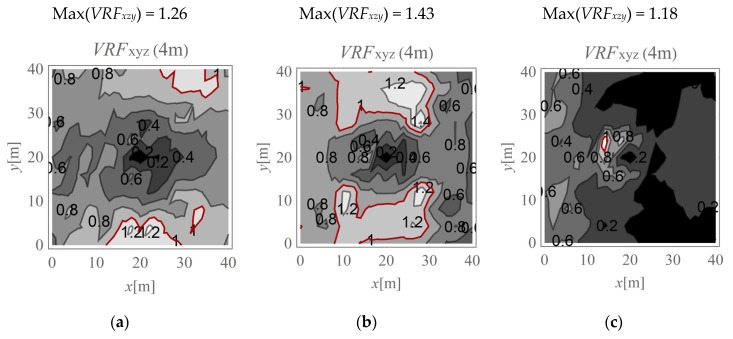
*VRF_xyz_* for the horizontal surface located at a depth of 4 m; (**a**) *G_xz_*_,1_ = *G_xz_*_,2_; (**b**) *G_xz_*_,1_ = 10*G_xz_*_,2_; (**c**) *G_xz_*_,2_ = 10*G_xz_*_,1_.

**Figure 13:**
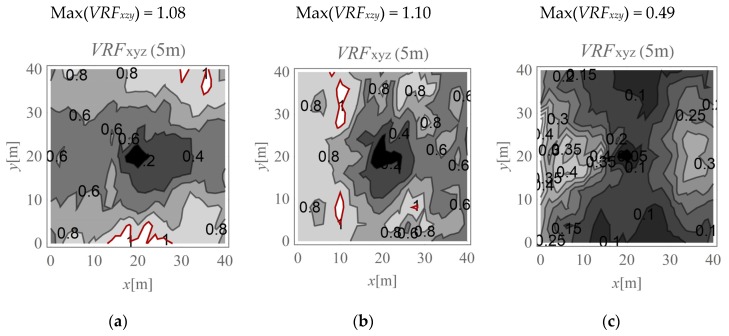
*VRF_xyz_* for the horizontal surface located at a depth of 5 m; (**a**) *G_xz_*_,1_ = *G_xz_*_,2_; (**b**) *G_xz_*_,1_ = 10*G_xz_*_,2_; (**c**) *G_xz_*_,2_ = 10*G_xz_*_,1_.

**Figure 14:**
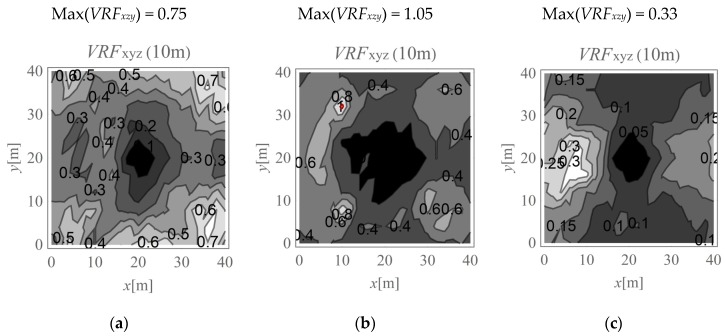
*VRF_xyz_* for the horizontal surface located at a depth of 10 m; (**a**) *G_xz_*_,1_ = *G_xz_*_,2_; (**b**) *G_xz_*_,1_ = 10*G_xz_*_,2_; (**c**) *G_xz_*_,2_ = 10*G_xz_*_,1_.

In the present study, the maximum value of the three velocity components is calculated for points located on the ground surface (*v_x,ho_, v_y,ho_*, and *v_z,ho_*) and at selected depths below it (*v_x,h_, v_y,h_*, and *v_z,h_*). The results are expressed according to Formula (4) (Figure 9, Figure 10, Figure 11, Figure 12, Figure 13 and Figure 14). The dimensionless velocity amplitude reduction factor (*VRF_xzy_*) is calculated for each selected point in the considered half-space and for its vertical projection on the horizontal boundary plane *h*_o_ = 0. The practical meaning of *VRF_xzy_* reflects how the maximum velocity component value measured at a point at a selected depth differs from that observed at a corresponding point located at the ground surface. To examine the possibility of wave amplification, different soil conditions are considered. A lower layer is assumed to appear at a shallow depth, and it is inclined in the *x*-direction, as shown in Figure 3. The soil parameters for each analysed case are summarised in Table 1. The lower layer has either larger (Case 3, *G_xz_*_,2_ = 10*G_xz_*_,1_) (Figure 9c, Figure 10c, Figure 11c, Figure 12c, Figure 13c and Figure 14c) or smaller (Case 2, *G_xz_*_,1_ = 10*G_xz_*_,2_) (Figure 9b, Figure 10b, Figure 11b, Figure 12b, Figure 13b and Figure 14b) shear stiffness compared to the upper layer. The results obtained for heterogeneous ground conditions are also compared to those for homogeneous ground conditions (Case 1, *G_xz_*_,1_ = *G_xz_*_,2_) (Figure 9a, Figure 10a, Figure 11a, Figure 12a, Figure 13a and Figure 14a). The red-line contours correspond to the reference value *VRF_xzy_* = 1.

The results of numerical investigations show that generally, with increasing depth, wave attenuation can be observed, and the wave energy spreads out. However, in many regions located at shallow depths (to a depth of 5 m), vibration amplification appears (*VRF_xzy_* > 1, Figure 9, Figure 10, Figure 11, Figure 12, Figure 13 and Figure 14). In the vicinity of the applied load, the results are not representative (due to the modelling error of the load transmission), so they are omitted here (white area in Figure 9 in the middle of the considered region). For example, in Figure 11b, the greatest amplification appears at points *x* ~ 30 m and *y* ~ 30 m (Max(*VRF_xzy_*) = 1.49). This result suggests that during the vibration monitoring of a structural element below the ground surface (e.g., a pipeline or tunnel) located in this area (*x* ~ 30 m and *y* ~ 30 m, or *x* ~ 30 m and *y* ~ 10 m), the velocity measured at the ground surface should be increased by approximately 50% to obtain the expected value below the ground surface. Only this increased value can be compared to the threshold values for underground structures proposed by different standards for vibration monitoring [10,11,12,13,14,15,16]. Generally, the greatest amplification effect may appear in the case when a stiff layer is located close to the ground surface (Figure 9b, Figure 10b, Figure 11b, Figure 12b, Figure 13b and Figure 14b). For such soil conditions, the maximum velocity components measured at depths of 3–4 m can be ≈50% greater than those at similar points located on the ground surface (same *x* and *y* coordinates) (Figure 11b and Figure 12b). When the boundary layer is close to the ground surface, the effect of wave amplification is observed to be more significant (Figure 9b, Figure 10b, Figure 11b and Figure 12b). For the points located in front of the applied force (*x* > 20 m), the effect of wave amplification below the ground surface is more significant compared that at the points located behind the force. The excavation site acts here, to a certain extent, as a wave trench sometimes used for vibration mitigation [42,43,44]; it induces a wave energy scattering effect, which is why the wave attenuation effect behind the applied force (*x* < 20 m) is more visible than that in front (Figure 9a, Figure 10a, Figure 11a, Figure 12a, Figure 13a and Figure 14a). When the lower layer has “better” mechanical characteristics than the upper layer (*G_xz,_*_2_ *=* 10*G_xz,_*_1_), the possibility of wave amplification below the ground surface is lower compared to that in the opposite case (*G_xz,_*_1_ = 10*G_xz,_*_2_) or for homogenous ground conditions (*G_xz,_*_2_ *= G_xz,_*_1_). Velocity amplification can generally be observed to a depth of 5 m. For the points located deeper than 5 m, this effect is less significant, and wave attenuation is dominant (Figure 13 and Figure 14).

## 5. Summary: Recommendations for Sensor Placement for the Vibration Monitoring of Structures

Based on the results of the numerical analyses presented in the previous section, the areas of the largest soil response are identified (Section 4.1 and Section 4.2), and recommendations for sensor arrangements in the case of horizontal excitation can be proposed. The following detailed guidelines for sensor location are proposed to avoid the risk of underestimating the damage potential of structures.

### 5.1. For Structures Located on the Ground Surface

- The maximal soil response is observed in the direction perpendicular to the direction of the applied force. Therefore, special attention should be paid to monitoring structures that lie on the line from the vibration source and in a direction perpendicular to the direction of excitation. Sensors should be located in this area.
- In the case of ceilings and rooves sensitive to dynamic loads, more attention should be paid to structures located in front of the excitation force (building 1), where much greater values (by approximately five times) of the vertical velocity components are generally measured compared to those in the opposite direction (building 2). In such a case, sensors should be located in the middle of the highest floor of a building located in front of the applied force to predict the largest dynamic response of the structural element.
- The closer the distance between the vibration source and structure is, the greater the measured value of the soil response. Sensors should be placed on the structural element (e.g., a wall or foundation) that is located closest to the vibration source.

### 5.2. For Underground Structures

- Special attention should be paid to cases in vibration monitoring when a “strong” soil deposit (e.g., dense sand, gravel) overlies a “weaker” one (e.g., silt, loam). For such subsoil conditions, the maximal soil response (*PPV*) below the ground surface can be ≈50% greater than the corresponding value measured on the ground surface. This effect is more significant in the region in front of the applied load and when the boundary surface between the two layers is located close to the ground surface. During vibration monitoring of underground structures in these soil conditions, special attention should be paid to the area in front of the applied load. When vibration monitoring is based on measurements made directly on the ground surface, the recorded values should be increased by approximately 50% compared with the threshold values. This approach can help to avoid underestimations of the structure’s damage risk.
- For homogenous ground conditions, the amplification of velocity amplitudes may also occur below the ground surface, but the effect is less visible compared to that in the case described above (a “strong” soil deposit overlies a “weaker” one). A practical conclusion is that for homogenous ground conditions, when the vibration monitoring of underground structures is based on measurements made directly on the ground surface, the recorded velocity amplitudes should be increased by approximately 40% compared with the threshold values. This issue is especially important for points located in the direction perpendicular to the direction of the force.
- The largest vibration amplification appears in the case when a “strong” soil deposit overlies a “weaker” one. The opposite soil layering (“weaker” soil layer located close to the ground surface) can also result in vibration amplification; however, the effect is less visible compared to that for homogeneous ground conditions and the case with the opposite layering. In the case of a “weaker” soil layer located close to the ground surface, vibration monitoring can be based on sensors located on the ground surface; however, before comparing recorded vibration amplitudes with threshold values, the recorded values should be increased by approximately 25% to avoid underestimations of the structure’s damage risk.
- The effect of vibration amplification can be observed to a depth of approximately 5 m. For points located deeper than 5 m, the velocity component values measured at the ground surface will generally be greater than those measured at the selected depths. Therefore, the vibration monitoring of underground structures located deeper than 5 m below the ground surface can be based on the measurements obtained by sensors located directly on the ground surface. The safety of a structure can be ensured with this approach.
- The largest differences between the soil responses measured below and on the ground surface appear at depths of 2–3 m for homogeneous ground conditions. For heterogeneous ground conditions, the largest amplifications can be observed deeper (3–4 m below the ground level), near where the interface between two layers appears. Therefore, the vibration monitoring of underground structures located at the mentioned depths (2–4 m) is particularly important.
- Obviously, the shorter the distance between the excitation force and an underground structure, the greater the measured velocities. Sensors should be located on or above the underground structure and as close as possible to the vibration source.

## 6. Conclusions

Structural health monitoring can be considered at different time scales, different scopes and with different objectives. Generally, structural health monitoring can be defined as ‘‘the process of implementing a damage identification strategy for aerospace, civil and mechanical engineering’’ [45] or “monitoring the structure through the evaluation of its in-service performance” [46]. According to Ostachowicz, structure health monitoring can be based on vibration-based monitoring, strain monitoring, elastic waves-based monitoring, electromechanical impedance-based monitoring, and comparative vacuum monitoring [46]. Although being in agreement with this general definition, the problem analysed in the paper is its simplified short-time version and should be distinguished from the structural health monitoring widely described in the literature [45,46,47,48]. Procedures of the parameter identification in structural health monitoring are based on the experimental-data considered and on the use of a numerical or analytical models for the problem description [47]. The aim is to find the material characteristics that give the model response as close as possible to the experimental outcomes. The idea of vibration monitoring of structures during geotechnical works is to measure a structure response to external dynamical excitation which happens in a close vicinity to a monitored object. The goal of vibration monitoring made during geotechnical works is to avoid structural damage; thus, measured values of a structure response are compared to threshold values defined by standards and codes [10,11,12,13,14,15] (Figure 2). When threshold values exceed the standards, geotechnical works have to be interrupted and design projects should be modified; if this does not happen, the probability of structural damage will increase or serviceability restrictions will be violated. The important issue of structural health monitoring is to specify appropriate sensor locations based on the optimization procedure [45,46,47,48] to obtain the most informative data. In the case of vibration monitoring during geotechnical works, the proper location of sensors is also of great importance for engineering practice: Sensors should be placed in critical locations where maximum structural response is expected and representative values are measured [10,11,12,13,14,15]. The goal of vibration monitoring during geotechnical works [10,11,12,13,14,15] is to predict damage before it appears in a structure, not to find its state or updated parameters.

In this paper, wave propagation caused by horizontal excitation in elastic subsoil is considered. The aim of the paper is to determine in which regions of the soil medium the maximum subsoil response may appear. This issue is important from the perspective of the vibration monitoring required for geotechnical works [49]. Sensor arrangement is crucial when considering the safety of structures and people [50]. This issue is especially important in the case of underground structures (e.g., pipelines and tunnels) when direct measurements of the structure’s response are not possible or unsafe to obtain. In many codes and standards for structure protection, threshold values for the *PPV* are usually presented for points located directly on underground structures [12,13,14]. The results presented in this paper provide the possibility of predicting the soil response below the ground surface based on measurements made directly at the ground surface. This paper provides guidelines for how the *PPV*s measured at the ground surface can be used to predict the *PPV*s that appear below the ground surface. In summary, from the presented investigations, the main conclusions for vibration monitoring in the case of horizontal impact excitation can be formulated as follows:

- Special attention should focus on the vibration monitoring of underground structures, which is usually based on measurements from sensors located directly on the ground surface. In such cases, the maximal values of the velocities that appear below the ground surface can be as much as 50% greater than those recorded on the ground surface. This effect is most significant in the case when a “strong” soil deposit overlies a “weaker” deposit and in the area located in front of the applied load. Before comparing the values measured on the ground surface with threshold values related to underground structures given by codes and standards, the recorded values should be increased. Thus, the risk of structural damage will not be underestimated.
- In the case of structures located at the ground surface, the largest soil response appears in the direction perpendicular to the excitation force direction (building 2). During the vibration monitoring of structures, focus should be placed on monitoring in this area. Notably, the shorter the distance to the vibration source is, the more significant the soil/structure response to dynamic excitation will be.

## Figures and Tables

**Table 1:** Dynamic properties of soil deposits for the three analysed cases.

		Dynamic Shear Modulus *G_xz_* [MPa]	Young’s Modulus in the Plane of Isotropy *E_x_* [MPa], *E_y_* = *E_x_*	Young’s Modulus in the Plane Perpendicular to the Plane of Isotropy *E_z_* [MPa]
Case 1	Deposit 1	*G_xz_*_,1_ = 30.0	*E_x_*_,1_ = 116.0	*E_z_*_,1_ = 85.9
	Deposit 2	*G_xz_*_,2_ = 30.0	*E_x_*_,2_ = 116.0	*E_z_*_,2_ = 85.9
Case 2	Deposit 1	*G_xz_*_,1_ = 300.0	*E_x_*_,1_ = 1160.0	*E_z_*_,1_ = 850.9
	Deposit 2	*G_xz_*_,2_ = 30.0	*E_x_*_,2_ = 116.0	*E_z_*_,2_ = 85.9
Case 3	Deposit 1	*G_xz_*_,1_ = 30.0	*E_x_*_,1_ = 116.0	*E_z_*_,1_ = 85.9
	Deposit 2	*G_xz_*_,2_ = 300.0	*E_x_*_,2_ = 1160.0	*E_z_*_,2_ = 850.9
